# Recurrence analysis of ant activity patterns

**DOI:** 10.1371/journal.pone.0185968

**Published:** 2017-10-09

**Authors:** Felipe Marcel Neves, Ricardo Luiz Viana, Marcio Roberto Pie

**Affiliations:** 1 Departamento de Zoologia, Universidade Federal do Paraná, Curitiba, Paraná, Brazil; 2 Departamento de Física, Universidade Federal do Paraná, Curitiba, Paraná, Brazil; Humboldt-Universitat zu Berlin, GERMANY

## Abstract

In this study, we used recurrence quantification analysis (RQA) and recurrence plots (RPs) to compare the movement activity of individual workers of three ant species, as well as a gregarious beetle species. RQA and RPs quantify the number and duration of recurrences of a dynamical system, including a detailed quantification of signals that could be stochastic, deterministic, or both. First, we found substantial differences between the activity dynamics of beetles and ants, with the results suggesting that the beetles have quasi-periodic dynamics and the ants do not. Second, workers from different ant species varied with respect to their dynamics, presenting degrees of predictability as well as stochastic signals. Finally, differences were found among minor and major caste of the same (dimorphic) ant species. Our results underscore the potential of RQA and RPs in the analysis of complex behavioral patterns, as well as in general inferences on animal behavior and other biological phenomena.

## Introduction

Animal behavior is characterized by broad differences in hierarchical organization and variability, from individual organisms to societies. The variability often detected in animal behavior seems unpredictable at first sight, resulting from genetic, developmental, neural and physiological processes, as well as environmental effects [1]. However, this apparent intrinsic variability could hide nonlinear and unstable deterministic signals. In a seminal work, Cole [1] observed that the time series of single, isolated workers of the ant *Temnothorax* (= *Leptothorax*) *allardycei* had movement activity characteristic of low-dimensional chaos, whereas periodic patterns emerge when larger numbers of ant workers are allowed to interact. Indeed, the existence of chaos in ant activity has been suggested as a necessary dynamic for foraging and exploration [2–3], yet empirical studies about the dynamics of activity (movement) patterns are still in their infancy (e.g. [1–2, 4–9]). Ants, in particular, display several advantages as a model organism for these kinds of study, given that they show considerable variation in social complexity (e.g. colony size, behavioral and genetic composition, and the way in which reproduction is partitioned among nestmates), morphological, ecological and behavioral traits at the colony-level [10–11]. Furthermore, ants are easy to manipulate and rear under laboratory conditions, and to observe in 2D experimental setups [10].

Activity could be considered as one of the most vital biological features of animals, being correlated with a wide spectrum of behavioral syndromes, such as aggressiveness and exploration [12–17]. A highly informative way to collect and investigate movement activity probably could be provided by the investigation of spatio-temporal data of several species. However, the study of time series patterns imposes some difficulties, not the least of which being data collection. A large amount of noise in data collection (as often occurs in real data, e.g. from EEG analysis, cardiology or geology) reduces the ability to detect deterministic signals. Fortunately, the possibility of automated tracking of individuals in recent years offers efficient ways to obtain qualitative reliable data [18]. Characterizing irregular behavior of deterministic or stochastic processes is not a straightforward task to perform either. Nevertheless, we can take advantage of tools from nonlinear dynamics based on the temporal component of the behavior to gain insights into its operation and evolution [19]. Several analysis methods have been proposed to investigate the presence of determinism in time series (e.g. Lyapunov exponents, Fourier analysis, Power spectral analysis), however, hitherto with very limited empirical applications [19].

In this study, we introduce the use of recurrence analyses for the study of animal behavior. Recurrence analysis is a new, reliable and robust method of nonlinear data analysis that could be used for an improved understanding of biological time series. It is composed of visual diagnostics known as Recurrence Plots (RPs), and measures of complexity, such as Recurrence Quantification Analysis (RQA) [20–21]. Using these tools, one can distinguish regimes of recurrence behavior, which may be characteristic of different processes, such as white noise, chaotic maps, and (quasi-) periodic processes [21]. RQA has several advantages when compared to other time series analysis, such as its mathematical simplicity, non-restrictive modelling assumptions, and the capacity to deal with inherent noise [19]. RQA has been used to interpret and correlate complex patterns in dynamic systems, such as in physics [22], physiology [23], meteorology [24], economics [25], geophysics [26] and cardiology [27]. The use of the RQA measures could give a more detailed and qualitative approach to time series analysis of complex dynamics. Here, we examine and compare the complex temporal pattern of movement activity dynamics of isolated individuals of species with varying levels of social complexity and behavioral specialization.

## Methods

Three ant species were used in our study: *Gnamptogenys striatula* (Ectatomminae), *Linepithema micans* (Dolichoderinae), and *Pheidole rudigenis* (Myrmicinae). Workers from three colonies of each chosen species were collected in the campus of the Universidade Federal do Paraná in Curitiba, state of Paraná, Brazil. *Gnamptogenys striatula* is typically found in open habitats and rainforests, showing a suite of primitive behavioral and morphological traits [28]. Colonies of *G*. *striatula* are small (150–200 individuals) and have either one or several queens and gamergates (i.e. workers with reproductive capacity) [29–30]. *Linepithema micans* belongs to a widespread genus that includes an important invasive species (i.e. *Linepithema humile* [31]), which could be an indicative of its own potential as an invasive species. Colony size in *L*. *micans* might exceed 1000 individuals, leading to a fairly complex social organization [32]. Finally, *P*. *rudigenis*, as is the norm for its genus, is characterized by a dimorphic sterile worker caste, with regular workers (minors) carrying out quotidian colony tasks, whereas larger, big-headed workers (majors) are specialized in specific tasks, such as colony defense or seed milling [33]. Furthermore, for comparison with non-social insects, we used adults of the beetle *Tenebrio molitor*, a cosmopolitan pest of stored grains with gregarious behavior. No specific permissions were required for the locations or activities reported in this manuscript. The study did not involve human participants, specimens or tissue samples, or vertebrate animals, embryos or tissues. Furthermore, the field studies did not involve endangered or protected species.

Ants were collected manually by attracting them outside their nests using sugar water or tuna baits between 10:30 am and 5:00 pm (*n* = 30 workers for each species, except for *P*. *rudigenis*, in which 30 minor and major workers were tested separately). Assays lasting for two hours (*n* = 7200 seconds) typically started 45 min after the collection, whereas *T*. *molitor* were reared in laboratory in three acrylic boxes, stored in a well-ventilated, dark place, at ambient humidity and temperature with food (i.e. wheat bran) *ad libitum*. Trials were carried with different combinations of colonies and species per day (i.e. at least 10 individuals for each colony; 30 individual time series by each species), for a total of 150 analyzed experiments (*n* = 300 hours). In order to extract the time series without the interference of fluctuating densities, only one single individual of each species was used in each trial, so that we only conducted analyses using isolated ants. Given that all chosen ant species have similar body sizes, we disregarded the possible effects of the body size of the individuals or its body parts when comparing the time series of each ant species. However, the adults of *T*. *molitor* are much larger than the ants (i.e. the ant species chosen have a mean body size of 2.6 mm while *T*. *molitor* has a body size of 15 mm). We justify the use of *T*. *molitor* in this study because we are not investigating the amplitude of the activity between the species, but how its dynamics, i.e. the changes of states between activity and inactivity over time occurs (the concept of activity considered in our study is explained in the section below).

### Experimental setup

**Fig 1:**
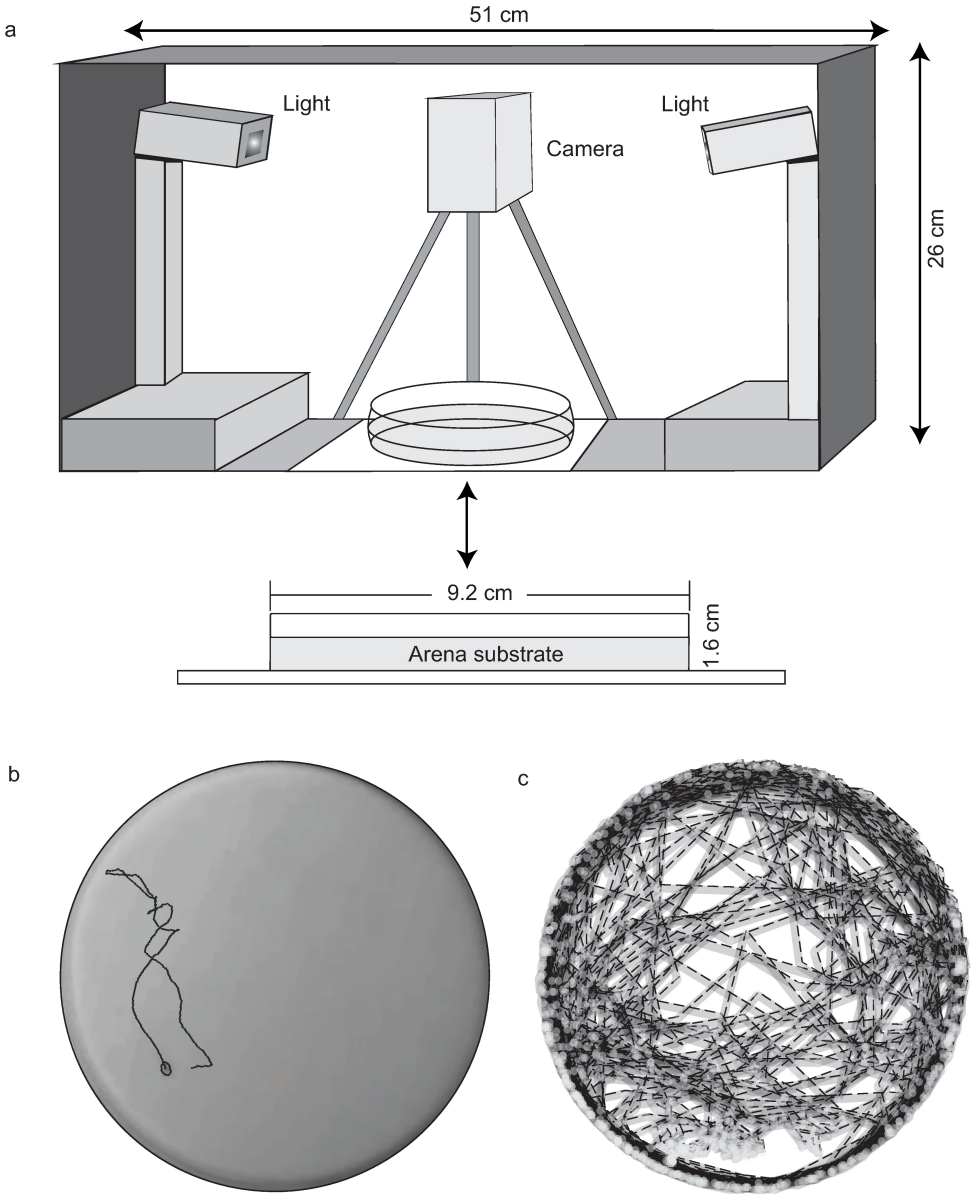
The experimental setup. Measurements, disposition view of the light spots, camera and the tracking arena (a). Video frame of an ant (*Linepithema micans*) in the arena during the tracking, the black line corresponds to the movement of the ant (b). Final tracking coordinates (x,y) of 2400 frames extracted from a time series (*Linepithema micans*), the grey dashed lines are the coordinates obtained (c).

The experimental apparatus for the trials consisted of an environmental chamber made with cardboard (51 x 26 cm) with a tracking Petri dish arena inside (92 mm in diameter) (Fig 1). The arena was brightly lit (≈880 lux) by two fluorescent light spots (Taschibra ^®^ TKT15 15W 120 VAC 60 Hz 370 mA 6.400 *K*) positioned at opposite corners. Individuals were placed inside the arena under controlled environmental conditions (21 ± 2°C and 65% ± 10 relative humidity), between a glass cover plate and the substrate, which was shallow enough to constraint movement into only two dimensions. The color of the floor of the arena was opaque white for better image contrast, consisting of odorless white rubber silicon RTV (i.e. Room Temperature Vulcanizing silicon CS1000). After each trial, both the arena and the substrate were cleaned, washed in bleach (80%), dried and not used for at least five hours before a new experiment.

We recorded the movements of the individuals with a camera mounted 20 cm above the experimental setup (Everio GZ-MG435BUB). Trials were recorded at 20 frames *per* second. The extracted raw movie (MPEG-2; 720x480 pixels) was transformed into uncompressed AVI video files by the program Virtual Dub software by Avery Lee, version 1.5.10. The Euclidean distance between the consecutive coordinates (x, y) of each frame, was considered the measure of activity (at an interval of three seconds; *n* = 2400 frames *per* trial). Thus, a high level of activity produced large number of pixel differences. Inactivity was characterized by the motionless state of the individual, which did not change its position within the interval of time considered between the frames. Activity was represented by white dots and inactivity by black dots in the RPs. Our definition of the activity measurement is similar to that of Cole [1] in the study on the activity behavior of the ant *Temnothorax albipennis*.

### Tracking system

The x-y coordinates of the individual positions were extracted using the Ctrax software (Caltech Multiple Fly Tracker; version 0.3.12) and the associated FixErrors toolbox for MATLAB (v. 7.10.0 2010; MathWorks, Inc., Natick, MA, USA [34–35]; MATLAB v. 7.10.0). Ctrax was primarily made for tracking fruit flies, but is able to track individuals and large groups of animals (e.g. cockroaches, fishes, ants) while maintaining their individual identities [35–37]. Furthermore, Ctrax has high accuracy in movement tracking and requires simple recording equipment [35]. During preliminary experiments, we observed false positives errors (type I) and false negative errors (type II). Type I errors (mismatches of object identity) were corrected manually with the help of the FixErrors toolbox available for the Ctrax software, whereas type II errors (loss of tracking) were accepted only when they accounted for less than 5% of the time series.

### Recurrence plots

We investigated the obtained time series using the Recurrence Plot (RP), which is a technique of nonlinear data analysis first proposed by Eckmann et al. [20]. RPs consists of a visualization (or a graph) of a square matrix in which the matrix elements correspond to those times at which a state in a dynamical system recurs, i.e. approaches itself after some period of time [20–21]. Consequently, the RP reveals all the times when the phase space trajectory of the dynamical system visits roughly the same region in the phase space [21]. We constructed recurrence matrices by comparing embedding vectors with each other at different times, drawing pixels when the distance between vectors falls within an *ε*-neighborhood [21, 38–39]. Such an RP can be mathematically expressed as

$${\text{R}}_{i,j}=H\left(\varepsilon -∥x_{i}–x_{j}∥\right),\;\left(i,j,=1,2\ldots N\right),$$

where *ε* is a threshold, *H*(.) is the Heaviside unit step function, ||…|| stands for some norm (e.g., the Euclidean norm), *i* = 1, 2,…*N* is represented in the horizontal axis, and *j* with the same range in the vertical axis. The RP is thus obtained by assigning a black (white) dot to the points for which **R**_*i*,*j*_ = 1(0). By construction, the recurrence matrix is always symmetric (**R**_*i*,*j*_ = **R**_*j*,*i*_), and a point is always recurrent to itself, i.e., **R**_*i*,*i*_ = 1 forming the main diagonal line of the RP [21].

Graphic representations of recurrence points permit to observe and interpret the general overview pattern of each individual time series. Eckmann et al. [20] made distinctions on how to visually read some of the plots. Marwan et al. [21] classifies recurrence plot structures into large-scale and small-scale patterns, Homogeneous patterns, characterizing white noise; Periodic patterns, when recurrence plots present diagonal lines and/or checkerboard structures; Drift patterns, when there is fading in the corners, for systems which are non-stationary; Disrupted patterns, for extreme (and rare) events, when white bands are present, indicating transitions. Furthermore, small-scale patterns can also be characterized, as in the case of isolated points, which is an expression of quickly changing/fluctuating states. Diagonal and vertical lines are important structural elements of RPs, creating the basis for its quantification. Diagonal lines appear when two parts of the phase space trajectory run parallel for some time, whereas vertical or horizontal lines occur when the system either does not change or it changes slowly [38]. Moreover, Zbilut and Webber [23] introduced measures based on the information extracted from the RPs to quantify dynamical features of the data. Recurrence quantification analyses (RQA) provide a characterization of the type of dynamics present in the system (e.g. periodic, chaotic) [21, 40]. RPs and RQA were obtained through the CRP toolbox (v. 5.17) developed by Norbert Marwan for MATLAB (MATLAB 7.10.0) [34]. The toolbox can be found at http://tocsy.pik-potsdam.de/CRPtoolbox/.

### Recurrence quantification analysis

RQA comprises many quantitative diagnostics of the distribution of dots (actually pixels) in a recurrence plot to provide quantification of important aspects revealed through the RPs in detail. We choose five measures to examine our data: Recurrence rate (RR), Determinism (DET), Entropy (ENT), Laminarity (LAM) and Trapping Time (TT). The recurrence rate (RR) is the probability of finding a black recurrence point (for which **R**_*i*,*j*_ = 1), or

$$\text{RR}=\frac{1}{N\left(N-1\right)}\Sigma_{i,j=1;i\neq j}^{N}\;R_{i,j},$$

where *N*^2^ is the total number of pixels (black or white) in a RP [21]. Recurrence rate uses the same definition of the correlation sum, which does not include the main diagonal line, being related with to probability that a specific state will recur. Higher RR (for the same value of the *ε* parameter) would indicate that there are only few overall changes in the dynamics of the responses over time and that performance is confined to few different states.

Determinism (DET) measures the percentage of points in an RP belonging to diagonal lines, indicating deterministic components in the recurrence plot [21]. The DET measure is calculated by,

$$\text{DET}=\frac{\sum_{l=l_{min}}^{l_{max}}lP\left(l\right)}{\sum_{i,j=1;\;i\neq j}^{N}R_{i,j}},$$

the *l*_*min*_ is the minimum and the *l*_*max*_ is the maximum length allowed for a diagonal line. *P*(*l*) = {*l*_*i*_; *i* = 1,2, …, *N*_*l*_} is the frequency distribution of the lengths *l*_*i*_ of diagonal lines, and *N*_*l*_ is the absolute number of diagonal lines, except for the main diagonal line [21]. The higher the DET value, more it reflects the predictability of the system over time. We can also compute estimates for the *Shannon entropy* (ENT),

$$\text{ENT}=-\sum_{l=l_{min}}^{l_{max}}p\left(l\right)l\text{n}\;p\left(l\right),$$

where

$$p\left(l\right)=\frac{P\left(l\right)}{\sum_{l=l_{min}}^{l_{max}}P\left(l\right)},$$

is the probability distribution of the diagonal line lengths. The ENT reflects the complexity of the deterministic structure present in a system [21]. Higher entropy would indicate more inherent complexity of the corresponding time series, e.g. for uncorrelated noise the value of ENT would be rather small, indicating its low complexity. Laminarity (LAM) is the percentage of RP points forming vertical lines, or of these laminar phases,

$$\text{LAM}=\frac{\sum_{v=v_{min}}^{v_{max}}vP\left(v\right)}{\sum_{i,j=1;\;i\neq j}^{N}R_{i,j}},$$

where *v*_*min*_ is the minimum lengths of a vertical line and *v*_*max*_ is the maximum vertical length. Analogously to diagonal lines, we can obtain the frequency distribution of the lengths *v*_*i*_ of vertical lines *P*(*v*) = {*v*_*i*_; *i* = 1,2, …, *N*_*v*_}, where *N*_*v*_ is the absolute number of vertical lines. LAM represents the occurrence of laminar states in the system without describing the length [21]. Moreover, we calculated the trapping time (TT) that is the average length of a vertical line, it’s given by

$$\text{TT}=\frac{\sum_{v=v_{min}}^{v_{max}}vP\left(v\right)}{\sum_{v=v_{min}}^{v_{max}}P\left(v\right)},$$

TT estimates the mean time that the system will remain at a specific state or how long the state will be trapped [21].

There are different ways into how to apply RQA, mostly based in order of magnitude (large and small scale) and data format (e.g. networks, time series). Here, we applied a global time series approach for each individual replicate. The global time series approach focuses on a large scale encompassing the entire time series with the five chosen RQA measures been extracted from it.

#### Recurrence parameters

The recurrence plots and the corresponding recurrence analysis used in the experiments might be affected by the chosen parameters from each time series and the embedding parameters affecting the quality of the phase space reconstruction, namely, time delay *τ*, embedding dimension *m* and the threshold value *ε*. The time delay *τ* determines the predictability of the components in the reconstructed vectors of the system state [21]. It should be chosen in a way such that the elements in the embedding vectors are no longer correlated. We estimate the time delay as the one where average mutual information reaches its first minimum [41]. The embedding dimension *m* determines the number of the components in the reconstructed vector of the system state. It should be large enough to unfold the system trajectories from self-overlaps, but not too large as the noise will be amplified. We employ the false nearest neighbor (FNN) method as suggested by Kennel et al. [42] to determine a good value for our system. The threshold value *Ɛ* was defined accordingly to each time series recurrence plot, it was chosen using the value that corresponds to 10% of the maximum phase space diameter of the data [20]. Through the methods exposed here, the recurrence measures were stipulated in such a way that the embedding dimension and the time delay for all the time series were defined with the value of three. The threshold value was defined individually according to each time series (i.e: threshold median value and interquartile range: *G*. *striatula* 0.71 (0.12); *L*. *micans* 0.74 (0.11); *P*. *rudigenis* minors 0.79 (0.16); *P*. *rudigenis* majors 0.74 (0.09); *T*. *molitor* 1.23 (0.29).

#### Surrogate data and statistical tests

We compared each time series original data with a shuffled surrogate, which is a common approach used for validation of results in time-series analysis [43]. In the context of RQA, significant differences between the RQA measures of the data and its surrogates could be indicative of a strong deterministic component (not deterministic in the mathematical sense, but meaning that the dynamics is not simply stochastic) with the absence of spurious elements present by the system, as well as indicating non-stationarity [21, 44]. It should be made clear that determinism in the RQA context actually reflects the predictability of the system over time. It is indeed possible for a stochastic process to produce such patterns in the RQA terminology [39], and one should not conclude that the process is “deterministic” in the usual sense. In order to create the surrogate time series, we shuffled the data *x*(*n*) randomly choosing a pair of points from the data chain and randomly exchanged the positions of such points for each trial. The procedure has been repeated *N* times, where *N* is the number of data points. This shuffling preserves the statistical distribution of the data (e.g. mean, variance) but destroys the phasic time-correlated information in the dynamics. This leads to an empirical distribution of the RQA measures under the null-hypothesis of independence in time and an identical distribution. Given that normality was not met for the RQA measures obtained based on a preliminary Lilliefors test [45], we used the two tailed Mann-Whitney non-parametric rank sum test for independence [46] to compare the recurrence measures between the original data for each species and it is corresponding shuffled data (with *p*≤0.05). The comparison between species was made by the Kruskal-Wallis test [47]; it is also a non-parametric one-way analysis of variance by ranks for testing equality of three or more population medians. The pairwise differences between the species were tested using the Dunn’s test (1964). The null hypothesis for each pairwise comparison is that the probability of observing a randomly selected value from the first group that is larger than a randomly selected value from the second group equals one half; this null hypothesis corresponds to that of the Wilcoxon-Mann-Whitney rank-sum test. Statistical analyses were performed by the R software v. 3.2.3 (R Core Team 2015 [48]) using the dunn.test package [49].

## Results

**Fig 2:**
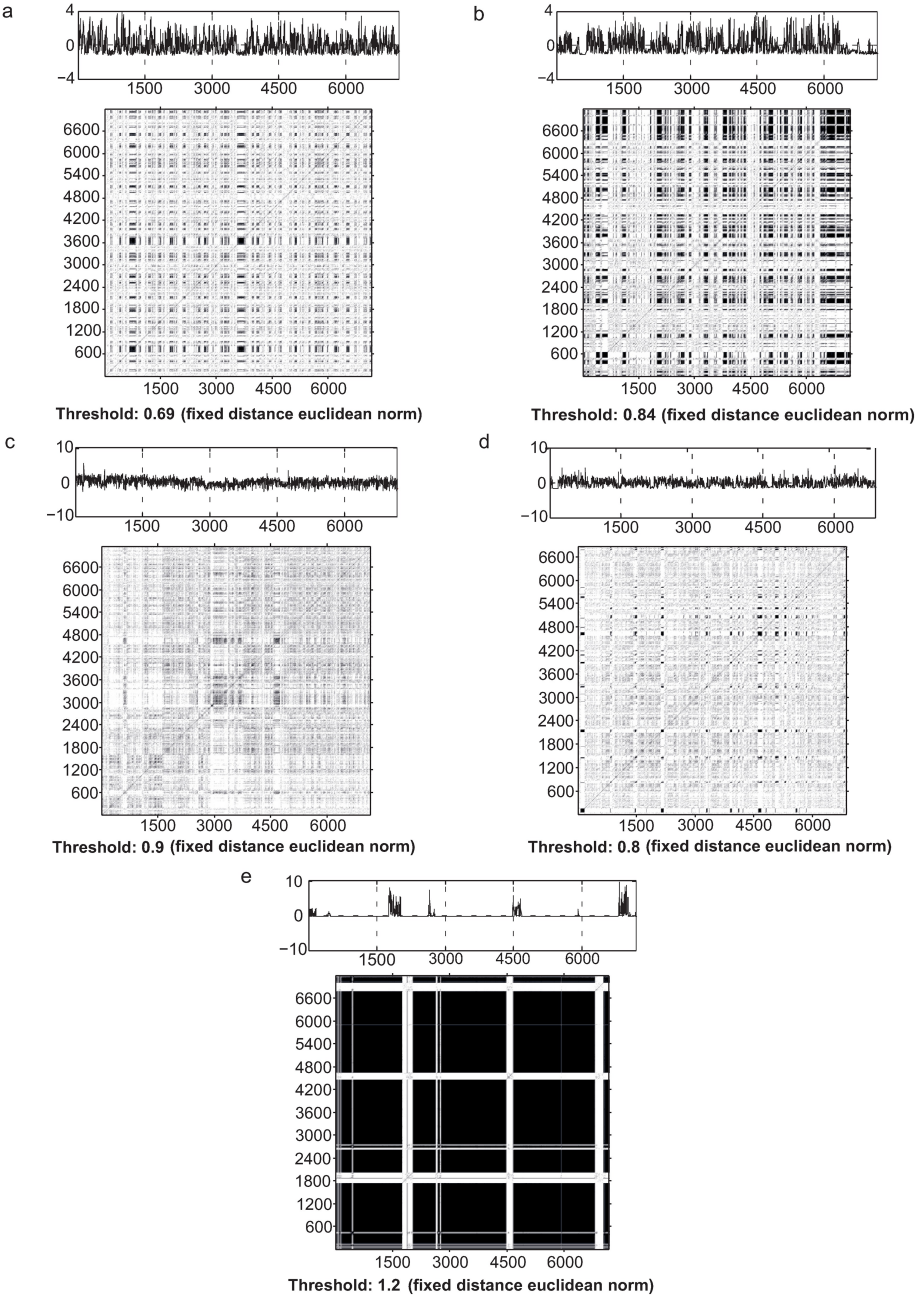
Recurrence plots (RP’s) of the time series. Each species had a characteristic recurrence plot pattern, here demonstrate by representative time series (approximately 2400 points; equivalent to 7200 seconds) with measures near the median RQA values from the replicates. The species are *Gnamptogenys striatula* (a), *Linepithema micans* (b), *Pheidole rudigenis* minor (c) and major subcaste (d), and *Tenebrio molitor* (e). In the RP’s, white dot are maximum distance and black dots are minimum. Estimated parameters: Dimension = 3, Time delay = 3 and the Threshold values were variable with each time series and are signalled in each RP.

We obtained a series of RQA global measures (*n* = 750) and individual RPs (*n* = 75) from a total of 150 time-series. The results highlight similarities and disparities between the activity dynamics of each species. Due to the topological nature of recurrence plots, we can also infer by visual inspection of RPs some of the features presented by all species and some particularities of them (Fig 2). All RPs showed several white bands, characterizing non-stationarity due to transitions. RPs showed diagonal and horizontal structures for almost all the species, supporting the hypothesis of a deterministic content present in the data. Furthermore, the RPs did not show homogeneous topologies, clearly rejecting the idea of a random process for most of the species. However, *P*. *rudigenis* had relatively short lines and isolated dots, indicating heavy fluctuation in the activity dynamics. This can imply the existence of uncorrelated random motions or even anti-correlated process. The RPs of *T*. *molitor* (Fig 2) have substantial phases of inactivity (black clusters) with a few sparse bouts of activity (white clusters), which is consistent with the influence of a deterministic quasi-periodic pattern.

We compared the global RQA measures of the original data matching the shuffled ones. In general, the surrogate data possess lower significant values of the RQA measures compared to the original data, with the exception of RR in *P*. *rudigenis* majors (Table 1). Differences between normal and surrogate data were usually higher in *G*. *striatula* and *L*. *micans*, with a typical percentage change between 38% and 86%. Differently, *P*. *rudigenis* and *T*. *molitor* had a percentage change of 7.3% to 88%. A higher percentage difference between the time series and their corresponding surrogates could indicate a more significant effect of the RQA values (i.e. higher differences of DET suggests a time series with strong predictable dynamics over time).

**Table 1:** RQA percentage measures of the species (columns) compared with each respective surrogate data series (_surr_). Recurrence rate (RR), determinism (DET), entropy (ENT), laminarity (TT) and trapping time (TT) of the original and surrogate data (_surr_).

DATA	*Gnamptogenys striatula*		*Linepithema micans*		*Pheidole rudigenis*_min_		*Pheidole rudigenis*_maj_		*Tenebrio molitor*	
RR	**0.08**	*p*<0.001	**0.11**	*p*<0.001	**0.08**	*p*<0.05	0.06	ns	**0.69**	*p*<0.001
RR_surr_	0.05	(38%)	0.05	(55%)	0.06	(25%)	0.04	(34%)	0.41	(41%)
DET	**0.51**	*p*<0.001	**0.45**	*p*<0.001	**0.31**	*p*<0.03	**0.28**	*p*<0.001	**0.97**	*p*<0.001
DET_surr_	0.09	(83%)	0.1	(78%)	0.08	(75%)	0.09	(68%)	0.65	(33%)
ENT	**1.43**	*p*<0.001	**1**	*p*<0.001	**0.82**	*p*<0.004	**0.79**	*p*<0.001	**2.57**	*p*<0.001
ENT_surr_	0.2	(86%)	0.19	(81%)	0.16	(81%)	0.17	(79%)	0.95	(64%)
LAM	**0.73**	*p*<0.001	**0.59**	*p*<0.001	**0.44**	*p*<0.04	**0.42**	*p*<0.001	**0.96**	*p*<0.001
LAM_surr_	0.16	(78%)	0.16	(73%)	0.16	(64%)	0.12	(72%)	0.89	(7.3%)
TT	**5.15**	*p*<0.001	**3.39**	*p*<0.001	**2.39**	*p*<0.003	**2.39**	*p*<0.001	**35.7**	*p*<0.001
TT_surr_	2.1	(60%)	2.12	(38%)	2.14	(11%)	2.09	(13%)	4.41	(88%)

The *p* value is based on the Mann-Whitney rank sum test for independent samples, it was considered significant when it was less than *p*<0.05. Significant results (represented by medians) are indicated in bold followed by the *p* value, non-significant (ns) results are also indicated. Moreover, results are followed by the percentage (%) difference between the normal and shuffled data (_surr_). The subscribed acronyms _min_ and _maj_ mean the words “minors” and “majors”, respectively.

RQA measures were also compared between species (Table 2). *Tenebrio molitor* presented significant higher RQA measures than the ants, however, these results alone must not be interpreted as a display of higher complexity. The ant species *G*. *striatula* and *L*. *micans* had in general significant RQA measures when compared with *P*. *rudigenis* (in the RR and DET values, only significant compared to the majors), whereas both *P*. *rudigenis* worker castes presented the lower results among all of the species. The worker castes of *P*. *rudigenis* differ significantly in the LAM values, where the *P*. *rudigenis* minors have higher RQA measures compared to the *P*. *rudigenis* majors (Table 2).

**Table 2:** Comparison of the RQA measures: Recurrence rate (RR), determinism (DET), entropy (ENT), laminarity (LAM) and trapping time (TT) between the species.

DATA	RR	DET	ENT	LAM	TT
*Gnamptogenys striatula*	0.08 (0.1)	**0.51** (0.44)	1.43 (1.13)	**0.73** (0.46)	**5.15** (4.5)
*Linepithema micans*	**0.11** (0.06)	0.45 (0.44)	1 (0.78)	**0.59** (0.37)	**3.39** (15.2)
*Pheidole rudigenis*_min_	0.08 (0.14)	0.31 (0.73)	0.82 (1.71)	**0.44** (0.68)	2.39 (10.9)
*Pheidole rudigenis*_maj_	0.06 (0.05)	0.28 (0.32)	0.79 (1.16)	0.42 (0.16)	2.39 (2.37)
*Tenebrio molitor*	**0.69** (0.3)	**0.97** (0.05)	**2.57** (0.50)	**0.96** (0.03)	**35.7** (42.7)

Pairwise differences were tested using Dunn’s test, as a post-hoc test after the Kruskal-Wallys test rank sum test. The results were indicated with each correspondent median and interquartile range (IQR), significant statistics results are indicated in bold (*p*<0.05). The subscribed acronyms _min_ and _maj_ mean the words “minors” and “majors”, respectively.

## Discussion

To the best of our knowledge, the present study provided the first application of recurrence analysis to the study of animal activity, as well as one of the first applications to biological phenomena outside the study of physiological and cardiac rhythms. The implications of our study are threefold. First, we found substantial differences between the activity dynamics of the gregarious beetles and the highly complex social ant species, with the results suggesting that the beetles have quasi-periodic dynamics and the ants do not. Second, workers from the different ant species varied with respect to their dynamics, presenting a degree of predictability as well as stochastics signals. Finally, differences were found among minor and major caste of the same (dimorphic) ant species. Each of these issues will be discussed in turn.

The activity pattern observed in the flour beetle *T*. *molitor* was characterized by the presence of periodic short bursts of activity, interspersed with longer periods of quiescence, as indicated by the extremely high values of vertical lines measures (LAM and TT). This could be also verified by the presence and consistency of wide laminar states of inactivity within the RPs (i.e. black clusters). The underlying causes of this behavior are still poorly known, but one possibility is that it is an involuntary response to the non-optimal conditions. In general, *T*. *molitor* is more inactive during daylight [50], normally evading natural light in search of darker areas [51]. The presence of long lines with different distances to each other in the RPs of *T*. *molitor* suggests a quasi-periodic dynamic (several frequencies in the system, whose ratios are irrational). Conversely, the time series of ants were more dynamic, composed by diagonal and horizontal structures from the RPs, RQA measures significantly higher in general than the shuffled time series but mixed with stochasticity (i.e. white-noise like pattern from the RPs, mostly present in *P*. *rudigenis*). Such patterns could be a result of noisy deterministic signals (i.e. in terms of predictability) or even low-dimensional deterministic chaos. Noise is extrinsic, it may come from various sources, such as uncertainties in the parameters of the system, fluctuations in parameters we do not have access (e.g. influence of the environment). Determinism is intrinsic, since it comes from the underlying dynamics of the system being studied. In biological systems, it is very likely that stochastic (noisy) behavior is always present, since there are always environmental factors acting on the system in an unpredictable and seemingly random fashion. One example would be the effect of the mutual interactions among the insects, which although of weak intensity, can affect randomly the behavior of individuals. Discriminating between noise and chaotic patterns in the RPs is not a trivial task [39]. Thus, here we limit to discuss what the presence of the observed patterns in the time series could mean in the context of ant behavior. Although the behavior of an organism may appear to be quite variable, the behavioral phenotype may be considerable less variable if a degree of predictability is present as we observed in the ant species. Since movement activity is closely related with other kinds of behavior, such as locomotion, patrolling (scouting), feeding or mating, all these behaviors may also display evidence of noisy deterministic signals. Furthermore, there is evidence from workers of the ant *Camponotus fellah* that single individuals are more active when isolated than at higher densities [52]. Some tasks, such as patrolling/scouting and foraging, which could be envisioned as information gathering processes outside the nest [53], also probably are composed by this kind of "hyperactive” behavior in isolated ants. Likewise, ants need to also respond to the cues provided by nestmates in a variety of contexts, from the recruitment during foraging to colony-level alarm behavior, for ensure the survival of the colony and its ergonomic efficiency [54–55]. Therefore, it is not surprising that ant activity patterns are more complex than those of solitary or even a gregarious species such as *T*. *molitor*.

There were interesting differences in activity patterns within the different ant species. For instance, both *L*. *micans* and *G*. *striatula*, which are monomorphic species, had higher RR and DET values. In monomorphic species, all colony tasks are performed by workers of equivalent morphology, with specialization only being possible through behavioral or age differences among workers [56]. On the other hand, in species with a dimorphic worker caste, some workers are morphologically adapted to specific tasks (e.g. colony defense, seed milling) whereas other workers can focus on more quotidian tasks, such as nest maintenance and brood care [53, 56–59]. Such differences are probably reflected in their intrinsic propensity to respond to specific cues in a way that is different from a colony with monomorphic workers. Indeed, in *P*. *rudigenis*, a species with polymorphic workers, both castes showed the lower RQA measures compared with the other species, with major caste presenting yet lower RQA values than the minor caste. Given that major workers in *P*. *rudigenis* probably play an important role in colony defense, the types of cues that they should respond are more stochastic or unpredictable in nature (e.g. encountering a forager from a competing colony or a predator). Thus, an activity behavior with a more unpredictable dynamic could be more adaptive given that randomness or chaotic behavior is an efficient response to environmental unpredictability, as explored in mathematical models based of ant behavior [3, 60–61]. Alternatively, the differences between castes could reflect the counterpart aspects of the intrinsic division of labor in a dimorphic species.

The search and interpretation of complex patterns in biological systems is an ambitious task and must be made with caution. Through our reductionist methodological approach using recurrence analysis, we propose the use of several RQA measures in conjunction with RPs, for a more consistent and comprehensive interpretation of the results. The possible use of recurrence analyses in the study of animal behavior are vast, from the interpretation of transitions within time series to the detection of synchronization and network complexity expect it to be an important tool in new empirical studies. Furthermore, the present study is among the first studies measuring individual complexity with comparison between species. The data generated by this kind of analysis could be interesting for behavioral ecologists as to physicists and correlated fields interested in the modelling and theoretical investigations of biological complex systems. The recurrence analysis permits further investigations to understand deeply patterns within time series. For instance, preliminary results indicate a progress to a more deterministic behavior with increasing densities, however by very different processes [62]. Our first study using recurrence plots and recurrence quantification analysis in animal behavior suggests that the activity dynamics of ants are composed by a plethora of complex patterns that ranges from stochastics signals and degrees of predictability.

## Supporting information

S1 File Dataset of the time series of movement activity. This dataset (Excel workbook format) is composed by the analyzed time series (normalized to zero-mean and standard deviation of one) of the activity dynamics of single individuals from all the species used in this study. The original x and y coordinates and non-normalized data are provided as well.
(ZIP)
